# Supplementary material for: Pfcrt copy number amplification detected in a Plasmodium falciparum outbreak
Source: bioRxiv. 2026 Jun 19:2026.06.18.732750. Preprint. [Version 1] doi: 10.64898/2026.06.18.732750 (PMC13308014; doi:10.64898/2026.06.18.732750)
Supplement: Supplement 2 [file media-2.pdf]

Supplementary Table 1. Within-sample diversity in Malaysia

FWS	Malaysia 2019	Malaysia 2010-2017
N	80	253
Mean	0.949	0.945
Median	0.956	0.949
Q1	0.952	0.944
Q3	0.958	0.953
N (FWS > 0.95)	63	114
N (FWS > 0.90)	76	249

Data was generated on 333 independent Malaysian *P. falciparum* samples using 116,704 biallelic SNPs.

Supplementary Table 2. Summary of genotype calls at drug resistance-associated variants in samples collected from the Philippines in 2013-2018.

Gene	Variant	Prop. Alt. Allele	No. Samples	No. Het.	Drug Assoc.	WHO Status
Pfcrt	47V>47M	2.56%	39	1		
	72C>72S	7.69%	39	3	CQ	Validated
	76K>76T	7.69%	39	3	CQ	Validated
	78F>78L	2.56%	39	1		
	220A>220S	2.50%	40	1		
	273H>273N	41.03%	39	10		
	280W>280R	2.56%	39	1		
	319F>319L	2.50%	40	1		
	326N>326D	7.50%	40	3		
	333T>333A	2.50%	40	1		
	356I>356L	2.56%	39	1	PPQ	Candidate
	371R>371G	2.56%	39	1		
	372E>372K	2.56%	39	1		
	378F>378S	2.56%	39	1		
Pfmdr1	86N>86Y	2.50%	40	0		
	90D>90G	2.50%	40	1		
	115C>115Y	2.50%	40	1		
	117D>117N	2.50%	40	1		
	130E>130K	7.50%	40	3		
	977E>977K	5.00%	40	2		
	1022R>1022G	2.50%	40	1		
	1025I>1025N	2.50%	40	1		
	1245R>1245K	5.41%	37	2		
	1261F>1261S	2.70%	37	1		
	1269I>1269V	2.78%	36	1		
	1277T>1277I	2.78%	36	1		
Pfk13	683I>683V	2.56%	39	1		

	678L>678M	2.56%	39	1		
	676A>676T	2.56%	39	1		
	671M>671I	2.56%	39	1		
	663L>663Q	2.56%	39	1		
	638G>638A	2.56%	39	1		
	567E>567G	2.50%	40	1		
	566V>566L	2.50%	40	1		
	515R>515G	7.50%	40	3		
	454V>454L	2.50%	40	1		
Pfdhfr	15C>15R	2.56%	39	1		
	20V>20I	2.56%	39	1		
	21E>21K	2.56%	39	1		
	25E>25K	2.56%	39	1		
	29N>29S	2.56%	39	1		
	54D>54N	2.50%	40	1		
	59C>59R	53.85%	39	8	PYR	Candidate
	59C>59Y	2.56%	39	1		
	68S>68L	2.56%	39	1		
	108S>108N	28.21%	39	4	PYR	Candidate
	109W>109R	5.13%	39	2		
Pfdhps	410E>410D	2.56%	39	1		
	421M>421V	2.56%	39	1		
	423N>423S	2.56%	39	1		
	424E>424K	2.56%	39	1		
	426A>426V	2.56%	39	1		
	437G>437A	76.92%	39	3	SP	Candidate
	461K>461E	2.56%	39	1		
	511P>511L	2.63%	38	1		

Samples with a minimum read depth ≥5 at variant position; Number of samples with a heterozygous call at the variant position (No Het). *Prop. Alt. Allele = Proportion of samples carrying an alternate allele with a read depth ≥5; No. Het. = Number of samples with heterozygous alleles at that position.

Supplementary Table 3. Combined results of realtime qPCR assay and *pfcrt* ONT long sequencing for Malaysian and Philippines samples.

Country	Sample	Realtime qPCR assay			PfCRT ONT Haplotype Sequencing			CN	Remark
		Avg FC	SD FC (range)	CN	Total depth	Estimated ratio over total depth	Haplotype		
	KF1	0.9844	0.0075 (0.9776-0.9912)	1	7281	0.827	H2	1	
	NKD4-101	0.9085	0.0181 (0.8920-0.9250)	1	973	0.793	H2	1	
	NKD4-111	-	-	-	35	0.343	H2	1	Low quality sample
	NKD4-115	0.9083	0.0147 (0.8949-0.9217)	1	4330	0.78	H2	1	
	NKD4-156	1.9462	0.0344 (1.9148-1.9777)	2					
	NKD4-167	0.9863	0.0106 (0.9766-0.9960)	1	5058	0.835	H2	1	
	NKD4-183	1.8358	0.0002 (1.8356-1.8360)	2					
	NKD4-23	1.9462	0.1130 (1.8430-2.0493)	2					
	NKD4-56	2.1851	0.0711 (2.1201-2.2500)	2					
	NKD4-64	1.0459	0.0021 (1.0440-1.0479)	1	6213	0.761	H2	1	
	QEM969	0.995	0.0022 (0.9930-0.9970)	1	8430	0.798	H1a	1	
	KF3	2.096	0.0205 (2.0772-2.1147)	2	7394	0.417	H1b	2	
						0.295	H2		
	NKD4-103	1.7494	0.0261 (1.7256-1.7732)	2	8371	0.379	H1b	2	
						0.373	H2		
	NKD4-114	-	-	-	37	0.333	H2	1	Low quality sample
	NKD4-118	-	-	-	41	0.354	H1b	2	
						0.463	H2		Low quality sample
	NKD4-154	1.7908	0.0546 (1.7410-1.8407)	2	528	0.4	H1b	2	
						0.438	H2		
	NKD4-179	1.8611	0.0150 (1.8475-1.8748)	2	5995	0.415	H1b	2	
						0.341	H2		
	NKD4-22	2.0152	0.2415 (1.7948-2.2356)	2	75	0.331	H1b	2	
						0.513	H2		Low quality sample
	NKD4-25	2.1313	0.0233 (2.1100-2.1526)	2	262	0.431	H1b	2	
						0.379	H2		
	NKD4-37	1.7587	0.0336 (1.7280-1.7894)	2	1759	0.374	H1b	2	
						0.38	H2		
	NKD4-43	1.9717	0.0310 (1.9434-2.0000)	2	55	0.291	H1b	2	
						0.455	H2		Low quality sample
	NKD4-70	1.8722	0.0151 (1.8584-1.8860)	2	4033	0.374	H1b	2	
						0.365	H2		
	NKD4-79	2.0411	0.1072 (1.9433-2.1390)	2	785	0.499	H1b	2	
						0.363	H2		
	NKD4-83	1.8655	0.1167 (1.7590-1.9721)	2	11385	0.378	H1b	2	
						0.292	H2		
	NQE4-28	1.9628	0.0315 (1.9340-1.9916)	2	5441	0.42	H1b	2	
						0.333	H2		
	QEM615	-	-	-	138	0.349	H1b	2	
						0.43	H2		
	QEM668	1.8389	0.0656 (1.7790-1.8988)	2	2594	0.377	H1b	2	
**Malaysia**						0.407	H2		
	PF-2013-03	0.955	0.0055 (0.9500-0.9600)	1	1292	0.854	H1b	1	
	Pf-2013-04	0.89	0.0219 (0.8700-0.9100)	1					
	Pf-2013-09	0.95	0.0329 (0.9200-0.9800)	1					
	Pf-2013-11	0.965	0.0712 (0.9000-1.0300)	1					
	PF-2013-17	0.905	0.0383 (0.8700-0.9400)	1	813	0.924	H1a	1	
**Philippines**	Pf-2013-28	0.92	0.0219 (0.9000-0.9400)	1					

	Pf-2013-30	1.12	0.2300 (0.9100-1.3300)	1					
	PF-2013-40	1.01	0.0438 (0.9700-1.0500)	1	170	0.191	H1a	1	Probably polyclonal; asymmetric haplotype ratio
						0.709	H1b		
	Pf-2013-57	0.895	0.2027 (0.7100-1.0800)	1					
	Pf-2013-61	0.985	0.0274 (0.9600-1.0100)	1					
	Pf-2013-73	1.01	0.0329 (0.9800-1.0400)	1	17	0.882	H1a	1	Low quality sample
	PF-2013-75	1.025	0.1150 (0.9200-1.1300)	1	125	0.904	H1a	1	
	PF-2013-78	0.925	0.0164 (0.9100-0.9400)	1	17697	0.869	H1a	1	
	PF-2013-83	1.05	0.0548 (1.0000-1.1000)	1	974	0.923	H1a	1	
	PF-2015-11	0.86	0.0000 (0.8600-0.8600)	1	281	0.826	H1b	1	
	PF-2015-12	0.92	0.0548 (0.8700-0.9700)	1	742	0.906	H1a	1	
	PF-2015-15	1.195	0.1917 (1.0200-1.3700)	1	56	0.875	H1a	1	Low quality sample
	PF-2015-16	0.995	0.0493 (0.9500-1.0400)	1	1413	0.89	H1b	1	
	PF-2015-18	0.86	0.0000 (0.8600-0.8600)	1	697	0.898	H1a	1	
	PF-2015-19	0.935	0.0822 (0.8600-1.0100)	1	178	0.362	H1a	1	single-copy pfcrt with minor sequence variation
						0.537	H1b		
	PF-2015-20	0.985	0.0383 (0.9500-1.0200)	1	1652	0.883	H1a	1	
	PF-2015-21	1.035	0.0164 (1.0200-1.0500)	1	40	0.9	H1a	1	
	PF-2015-22	0.935	0.0712 (0.8700-1.0000)	1	462	0.92	H1a	1	
	PF-2015-39	0.72	0.0767 (0.6500-0.7900)	1	15	0.933	H1a	1	Low quality sample
	PF-2015-42	1.08	0.1315 (0.9600-1.2000)	1	17	0.882	H1a	1	Low quality sample
	PF-2015-43	1.105	0.2136 (0.9100-1.3000)	1	5931	0.897	H1b	1	
	PF-2015-44	1.015	0.0931 (0.9300-1.1000)	1	227	0.899	H1a	1	
	PF-2015-46	0.965	0.0602 (0.9100-1.0200)	1	2542	0.852	H1a	1	
	PF-2015-48	0.995	0.0164 (0.9800-1.0100)	1	40	0.9	H1a	1	Low quality sample
	PF-2015-49	1.085	0.1260 (0.9700-1.2000)	1	113	0.823	H1b	1	
	PF-2015-50	-	-	-	24	0.917	H1a	1	Low quality sample
	Pf-2015-58	1.095	0.1807 (0.9300-1.2600)	1					
	PF-2015-59	1.05	0.1095 (0.9500-1.1500)	1	229	0.572	H1b	1	
	PF-2015-76	0.98	0.1643 (0.8300-1.1300)	1	50	0.94	H1a	1	
	PF-2015-80	1.105	0.1917 (0.9300-1.2800)	1	112	0.821	H1b	1	
	PF-2015-81	0.895	0.0055 (0.8900-0.9000)	1					Low quality sample
	PF-2015-82	1.13	0.1862 (0.9600-1.3000)	1	1644	0.918	H1a	1	
	PF-2017-18-36	-	-	-	942	0.914	H1a	1	
	PF-2018-15	1.04	0.0657 (0.9800-1.1000)	1	2697	0.916	H1a	1	
	Pf-2018-28	1.135	0.2574 (0.9000-1.3700)	1					
	PF-2018-37				2382	0.903	H1a	1	
	PF-2018-52				9623	0.901	H1a	1	
	PF-2018-60	0.965	0.0164 (0.9500-0.9800)	1	62	0.903	H1a	1	Low quality sample
	PF-2018-64	0.93	0.0329 (0.9000-0.9600)	1	3729	0.894	H1a	1	
	Pf-2018-8	0.92	0.0219 (0.9000-0.9400)	1					
	PF-2013-72	2.11	0.0329 (2.0800-2.1400)	2	674	0.591	H1b	2	
						0.305	H3		
	PF-2015-47	1.95	0.1643 (1.8000-2.1000)	2	165	0.255	H1a	2	
						0.639	H2		
	QEM192*	2.0389	0.1084 (1.9400-2.1379)	2	5112	0.32	H1b	2	
						0.287	H4		

*Imported case to Malaysia (2011), with travel history to the Philippines.

Supplementary Table 4. Summary of the demography of Malaysian 2019 outbreak cases.

Site	Country	Collection period	Age category (years)			% Male patients
			<5	5-15	>15	
Sabah	Malaysia	Jan 2019 - June 2023	11/109 (10.09%)	61/109 (55.96%)	37/1409 (33.94%)	62/109 (56.88%)

Supplementary Table 5. TaqMan *pfcrt* copy number quantitation assay primer and probe sequences and *pfcrt* ONT sequencing primers

Primer label	Sequences (5’-3’)
Pfcrt_TaqMan_F	ACGACACCGAAGCTTTAATTTAC
Pfcrt_TaqMan_R	TTTCCAGTAGTTCTTGTAAGACCT
Pfcrt_TaqMan_Probe	TGCTATATCCATGTTAGATGCCTGTTCAGT
B-tubulin_Taqman_F	CCCATTCCCACGTTTACATTTC
B-tubulin_Taqman_R	GGCACAGTTAAGGCTCTGTAT
B-tubulin_Taqman_Probe	CGGGTTTGCTCCTTTAACTAGTAGAGGC
Pfcrt_ONT_F	CCGTTAATAATAAATACACGCAG
Pfcrt_ONT_R	TCCTTATAAAGTGTAATGCGATAGC
